# INCREMENTAL COST AND READMISSIONS ASSOCIATED WITH COLECTOMY PATIENTS UNDERGOING HOME HEALTH SERVICES

**DOI:** 10.1093/geroni/igz038.2684

**Published:** 2019-11-08

**Authors:** Neil Kamdar, Elham Mahmoudi

**Affiliations:** 1 University of Michigan, Ann Arbor, Michigan, United States; 2 University of Michigan, Department of Family Medicine, Ann Arbor, Michigan, United States

## Abstract

Home health services has been considered a recommended form of post-discharge management of older patients undergoing surgery. In this study, we examined total 30-day episode payments and 30-day readmission rates for those who were and were not utilizers of home health services following colectomies. We utilized administrative claims data (2012-2017) from the Michigan Value Collaborative across 77 hospitals in Michigan. Post-acute care includes skilled nursing facility, inpatient/outpatient rehabilitation, emergency department visits, home health services, and other outpatient visits. All payments were risk adjusted using generalized linear models using Hierarchical Condition Categories, age, sex, insurance type, and prior 6-month payments. Controlling for selection bias for those utilizing home health services, we performed propensity score matching at a caliper of 0.001 without replacement. We identified 15,531 colectomies with 4,397 (28.3%) utilizing home health services within 30 days. There were 4,197 home health users and non-users with home health users having significantly higher adjusted 30-day episode payments ($33,254 vs. $30,463, 95% CI Diff: ($2,097, $3,486)). While there were only slight differences in adjusted index surgical base payments between home health users ($21,531, 95% CI: ($21,279, $21,783) and non-users ($20,703, 95% CI: ($20,409, $20,998)), there were substantially larger post-discharge payments for home health users ($3,617 vs, $1,839, 95% CI Diff: ($1,597, $1,959)). Colectomy patients utilizing home health services had a higher readmission rate (21.5% vs. 15.6%, p <0.0001). Colectomy patients utilizing home health services are more resource intensive in the post-discharge period. Recommendation for home health services increases costs and readmission.

